# Contrasting patterns of overweight and thinness among preschool children of different ethnic groups in Norway, and relations with maternal and early life factors

**DOI:** 10.1186/s12889-018-5952-1

**Published:** 2018-08-23

**Authors:** Ingun Toftemo, Anne Karen Jenum, Per Lagerløv, Pétur B. Júlίusson, Ragnhild Sørum Falk, Line Sletner

**Affiliations:** 1 Department of General Practice, Institute of Health and Society, University of Oslo, Postboks 1130 Blindern, N-0318 Oslo, Norway; 2 General Practice Research Unit (AFE), Department of General Practice, Institute of Health and Society, University of Oslo, Postboks 1130 Blindern, N-0318 Oslo, Norway; 3 Department of Clinical Medicine, Section of Paediatrics, University of Bergen, N-5021 Bergen, Norway; 4 Oslo Centre for Biostatistics and Epidemiology, Oslo University Hospital, PB 4950 Nydalen, 0424 Oslo, Norway; 5 Department of Child and Adolescent Medicine, Akershus University Hospital, Lørenskog, Norway

**Keywords:** Ethnicity, Preschool, Overweight, Obesity, Thinness, BMI

## Abstract

**Background:**

Childhood obesity is a worldwide health challenge and risk factor for adult life obesity, which predisposes to development of type 2 diabetes and cardiovascular diseases. However, also thinness in early life has been related to these diseases, especially if followed by fat gain. In European countries, susceptibility to cardio-metabolic diseases varies considerably between ethnic groups. We investigated ethnic differences in overweight and thinness in a multi-ethnic, population-based cohort of preschool children in Norway, and associations with maternal and early postnatal factors.

**Methods:**

Participants were children aged 4–5 years (*n* = 570) drawn from the population-based STORK Groruddalen cohort of healthy women and offspring followed from early pregnancy. Ethnic groups were: European (*n* = 298), South Asian (*n* = 154), and Middle East/North African (*n* = 118). Children’s growth data were provided from routine visits at local Child Health Clinics. Weight status was defined by the International Obesity Task Force. Using multinomial logistic regression analysis, we explored ethnic differences in overweight and thinness, and associations with maternal-, pre, − and postnatal factors.

**Results:**

Children of Middle East/North African origin had higher prevalence of overweight (22.0%) compared to European (12.8%) children, and in adjusted logistic regression analysis almost the double risk (OR 1.98; 95%CI: 1.08–3.63). Prevalence was lower in children of South Asian origin (5.2%). Children with South Asian background had higher prevalence of thinness (26.0%) compared to ethnic Europeans (10.4%), and the double risk (OR 2.20; 95%CI: 1.25–3.87) in adjusted models. Applying newly suggested BMI adjustments in South Asian children, taking into account their relatively increased adiposity, markedly increased the prevalence of overweight, and decreased the prevalence of thinness in this subgroup. Birthweight and maternal prepregnant overweight were strongly, positively associated with overweight, and inversely associated with thinness. Lower maternal age was associated with overweight only.

**Conclusions:**

In a multi-ethnic cohort we found strikingly different patterns of overweight and thinness among children of different ethnic groups at age 4–5 years, and a strong association between maternal BMI and their children’s weight status. More knowledge is needed on what characterizes and what promotes healthy growth patterns in multi-ethnic populations.

**Electronic supplementary material:**

The online version of this article (10.1186/s12889-018-5952-1) contains supplementary material, which is available to authorized users.

## Background

An increase in the prevalence of overweight and obesity among preschool children has been widely reported over the last decades. This represents a serious public health challenge [1]. Currently, the majority of children with overweight live in low- and middle-income countries where the overall prevalence is still increasing [1]. In European preschool children, the prevalence of overweight seems to have reached a plateau during the last years, although the prevalence varies considerably between countries. The rates tend to be higher in girls than in boys [2–4]. Findings indicate that some ethnic minority children with Middle East or North-African origin may be disproportionally affected by childhood overweight [4, 5], while other ethnic minorities, such as South Asian children, may have lower risk [6].

Childhood overweight and obesity have been associated with maternal factors such as prepregnant overweight and obesity, low socioeconomic position, and gestational diabetes mellitus [7–9]. Further, high birth weight and rapid weight gain in infancy also increase the risk [9]. Childhood overweight is a strong predictor for adult obesity and increases the risk of insulin resistance, type 2 diabetes, and cardiovascular diseases [10, 11].

However, cohort studies with long-term follow-up indicate that thinness at birth and early childhood may also be a risk factor for type 2 diabetes and cardiovascular disease, in particular if followed by an increase in body mass index (BMI) later in life [12–15]. This growth pattern most likely represents low lean mass from birth, which tracks throughout adult life, followed by accumulation of fat mass later in life. This “thin-fat phenotype” increases the risk of later cardio-metabolic diseases, but is not necessarily linked to adult overweight as defined by a high BMI [15–17]. Some ethnic groups, particularly South Asian populations, have an increased risk for type 2 diabetes and cardiovascular disease for a given BMI compared to populations with European or African origin. The susceptibility of South Asians is believed to be partly explained by their predisposition to a thin-fat phenotype already from birth [13, 18, 19]. By measuring body fatness in a multi-ethnic population of children in UK, Hudda et al. showed that BMI underestimated body fat in South Asian children, and suggested a positive adjustment of BMI of + 1.12 kg/m^2^ to account for greater relative adiposity in this population [20].

Pregnancy, the postpartum period, and early childhood are increasingly recognized as underused windows of opportunity to improve public health in the short and longer term [21]. In Norway, as in many other countries, pregnant women and their children are followed up regularly at Child Health Clinics. Increased knowledge about factors influencing overweight and thinness in multi-ethnic child-populations could contribute to an improved identification of children at risk, who could be offered targeted interventions in early life with the potential to reduce ethnic and social differences in health [22]. The aim of the current study was to investigate ethnic differences in overweight and thinness in a multi-ethnic, population-based cohort of preschool children in Norway, and associations with maternal and early postnatal factors.

## Methods

### Design and study population

The data were drawn from a prospective, population based cohort study, STORK Groruddalen, of 823 healthy pregnant women living in North-East Oslo [23]. Pregnant women were eligible for the study if they were 1) living in one of 3 city districts in Groruddalen; 2) planning to give birth at one of the 2 study hospitals; 3) at ≤20 weeks’ gestation; 4) not having pre-pregnancy diabetes or other diseases requiring intensive hospital follow-up during pregnancy; 5) able to communicate in Norwegian or any of the 8 languages to which all the information materials and questionnaires were translated (Arabic, English, Sorani, Somali, Tamil, Turkish, Urdu and Vietnamese); and 6) able to give informed written consent. While attending prenatal care at the Child Health Clinic, women were enrolled at mean 15 weeks of gestation, from 2008 to 2010. The Groruddalen area has a population that covers a large span in socioeconomic status, and has a high proportion of ethnic minorities. Of the study population, 59% had ethnic minority background. Questionnaire data were collected by specially trained midwifes through interview, supported by a professional interpreter when needed.

### Outcome variable

Virtually all children in Norway attend a Child Health Clinic regularly for vaccinations and check-ups. For this study we used children’s growth data from the routine preschool visit performed at age 4–5 years at the local Child Health Clinic. Trained child health care nurses measured weight, to the nearest 100 g and height, to the nearest 0.1 cm. BMI was calculated as weight/height^2^ (kg/m^2^). Our outcome variable was weight status at age 4–5, classified as an ordinal variable with 4 levels: “thinness”, “normal weight”, “overweight” and “obesity” using the age- and sex-specific BMI cut-off values (z-scores) defined by the International Obesity Task Force [24]. These cut-off values correspond to centile curves passing through BMI 18.5 (thinness grade 1), 25 (overweight) and 30 (obesity) at age 18 years. In the regression analysis, the overweight and obesity group were merged and referred to as “overweight”.

### Exposure variables

We chose exposure variables according to the literature and availability. The following variables were collected by interviews and questionnaires at inclusion:

*Ethnicity* was considered the main exposure variable and defined by the child’s mother or maternal grandmother’s country of birth if this country was outside Europe. We had information on 81% of fathers. Based on these data and clinical experience, there were very few children of mixed ethnicity (< 5% of ethnic European, South Asians and children with Middle East / North African origin). For this study we chose to focus on results for 3 main ethnic groupings: 1) Europeans (primarily from Norway and other Scandinavian countries); 2) South Asians (primarily from Pakistan and Sri Lanka); 3) Middle East/North Africans (primarily from Iraq, Turkey, Morocco, Afghanistan, Somalia, and Ethiopia).

*Mother’s pre pregnant BMI* was used as a categorical variable based on measured height and self-reported pre-pregnancy weight at inclusion [23], divided into three levels: Thinness (BMI < 18.5 kg/m^2^), normal weight (BMI 18.5–25 kg/m^2^) and overweight including obesity (BMI > 25 kg/m^2^).

*Education* was defined as an ordinal variable: Lower level (primary education or less), middle level (completed high school/upper secondary), and higher level (completed university/university-college education ≥4 years).

*Prepregnant physical activity* was self-reported and collected by a questionnaire that is previously validated against a physical activity monitor [25]. This variable is defined as moderately intensive activity for 30 min for ≥5 days/week, moderately intensive activity for 2.5 h/week over ≥3 days, vigorous-intensity activity for ≥20 min 3 times per week, or activity of both moderate and vigorous intensity (e.g., vigorous activity once per week and moderate activity twice per week). Prepregnant physical activity < 1 year prior to pregnancy was coded as *never.* Prepregnant physical activity > 1 year prior to pregnancy was coded as *regular*.

*Mother’s dietary patterns* were collected in week 28 of pregnancy, through a food frequency questionnaire, and defined by cluster analysis on 55 variables of intake and dichotomized into healthy and unhealthy [26].

*Gestational diabetes mellitus* was for this study defined according the WHO_2013_ criteria; fasting glucose ≥5.1 mmol/l and/or 2-h plasma glucose ≥8.5 mmol/l. A glucose tolerance test was performed in gestational week 28.

#### Other variables

Age at enrolment was used as continuous variable, and parity dichotomized as nulliparous and parous. Child birthweight to the nearest gram was routinely collected at birth on calibrated electronic scales [27]. Breast feeding was based on self-reports at 14 weeks postpartum and categorized as any and no breastfeeding. Maternal smoking status was also considered as covariate, but as very few of the ethnic minority women smoked, this variable was left out from the analyses.

#### Sample size

**Fig. 1:**
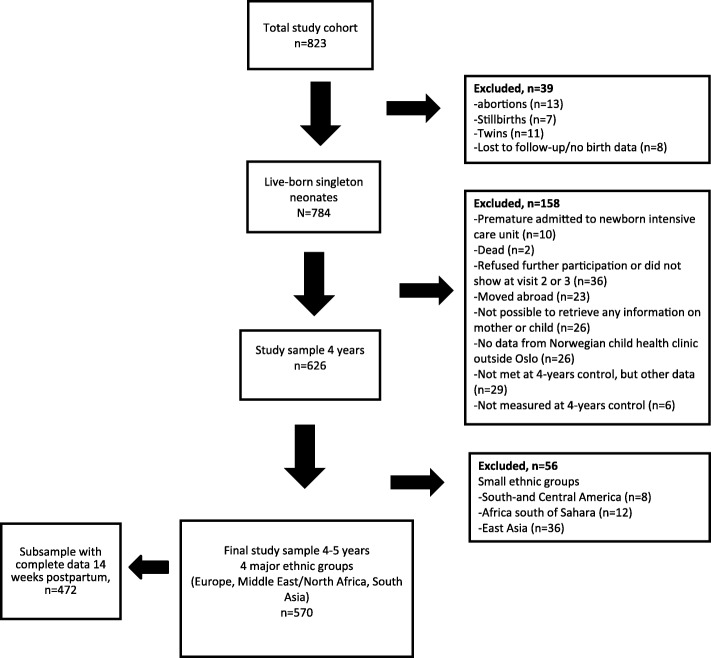
Flow chart

Of 823 women enrolled in early pregnancy, 784 singleton live born neonates with valid birth data were born (Fig. 1, flow chart). Of these neonates, 158 were excluded due to prematurity, postnatal death, refused further participation, family moved abroad or mother changed social security number, no data from Child Health Clinics outside Oslo, or missing data at Child Health Clinic at age 4–5 years. At last, 56 children from smaller and heterogeneous ethnic groups (East Asia, South- and Central America, and Sub- Saharan African countries) were excluded. The final study sample consisted of 570 children from 3 ethnic groups; 298 (52.3%) ethnic Europeans, 154(27.0%) ethnic South Asians, and 118 (20.7%) with Middle East/North African ethnicity.

### Statistical analysis

Descriptive statistics are given as frequencies, proportions (%), and means with standard deviation (SD). Differences in prevalence of thinness and overweight between the ethnic groups were assessed by Chi-square tests. Differences in children’s age were assessed by one-way ANOVA tests.

To investigate the impact of maternal and postnatal determinants on the child’s risk of overweight and thinness at the age of 4–5 years, we performed multinomial logistic regression analysis. Overweight and thinness were compared with normal weight children. Ethnic origin was the main exposure variable of interest (Europeans as reference). The following variables were considered potential covariates; maternal education, parity, age, prepregnant BMI and physical activity, diet during pregnancy, gestational diabetes mellitus, and child sex and birth weight. We selected variables into the multivariate model by a purposeful selection approach [28]. Variables with a *p*-value < 0.2 in the univariate analysis were included into the multivariate model. We removed one variable at a time, the variable with the highest p-value first, until all variables reached the level of significance (*p* < 0.05) in at least one of the models. For each step we checked if our main effect estimates (ethnicity) changed more than 15%. If so, the variable was kept in the model to take into account the potential confounding effect on the outcome. In the final model we tested for interactions with ethnicity by entering cross-product terms one-by–one. No significant interactions were observed. Results are shown as odd ratios (ORs) with 95% confidence intervals (CI). We included breast feeding into the multinomial logistic regression analysis of a subgroup with complete data at 14 weeks postpartum.

As it has been reported that children with South Asian origin have a higher fat mass for a given BMI than the other ethnic groups, we also performed positive BMI adjustments of + 1.12 kg/m2 for this group [20]. We then made a sensitivity logistic regression analysis using this adjusted classification of weight status for South Asians as outcome variable.

For all analyses we used SPSS version 22 (IBM SPSS statistics, NY, USA).

## Results

### Characteristics

Compared with European women, ethnic minority mothers were younger, had lower education, and higher parity. They were less physically active before pregnancy, and smoked less (Table 1). Prepregnant BMI was lower in South Asian women and higher in women from Middle East/North Africa. The prevalence of gestational diabetes mellitus was higher in ethnic minority mothers, and more minority women had a less healthy diet compared to ethnic European mothers. European children had higher birthweight than children in the other ethnic groups.

**Table 1:** Baseline characteristics

Variables	Ethnic origin n (%)			
	Total	Europe	Middle East/North Africa	South Asia
	*n* = 570	*n* = 298 (52.3)	*n* = 118 (20.7)	*n* = 154 (27.0)
	n (%)	n (%)	n (%)	n (%)
Mothers				
Maternal age, mean (SD)	29.9 (4.8)	30.7 (4.5)	29.3 (5.5)	27.8 (4.5)
Prepregnant BMI, kg/m2 (SD)	24.5 (4.8)	24.6 (4.7)	26.1 (5.7)	23.7 (4.3)
Prepregnant weight status				
Thinness, BMI < 18.5	30 (5.4)	12 (4.1)	6 (5.1)	12 (7.9)
Normal weight, BMI 18.5–25	313 (55.9)	170 (58.2)	52 (44.4)	91 (60.3)
Overweight, BMI > 25	217 (38.8)	110 (37.7)	59 (50.5)	48 (31.8)
missing	10	6	1	3
Nulliparous	267 (46.8)	161 (54.0)	41 (34.7)	65 (42.2)
Education				
Primary education or less	95 (16.8)	15 (5.1)	52 (44.1)	28 (18.5)
Completed high school/upper secondary	214 (37.8)	90 (30.5)	47 (39.8)	77 (50.3)
Completed university/college education	257 (45.4)	190 (64.4)	19 (16.1)	48 (31.4)
missing	4	3	0	1
Prepregnant regular physical activity^a^, *n* = 570	174 (30.6)	132 (44.3)	18 (15.3)	24 (15.6)
Gestational diabetes mellitus^b^, *n* = 556	180 (32.4)	72 (24.7)	41 (36.3)	67 (44.1)
Non-healthy diet^c^, *n* = 551	86 (15.6)	15 (5.2)	27 (25.0)	44 (28.9)
Smoking 3 months before pregnancy, *n* = 478	104 (21.8)	90 (32.1)	11 (12.5)	3 (2.7)
Children				
Sex, boy (%)	284 (49.8)	157 (52.7)	52 (44.1)	75 (48.7)
Birth weight (mean), g (SD)	3431.1 (548.6)	3577.9 (527.1)	3413.4 (509.5)	3226.1 (526.8)
Subsample with data 3 months postpartum				
Breast feeding 14 weeks postpartum, *n* = 472	404 (85.6)	214 (84.9)	80 (88.9)	110 (84.6)

^a^ Self-reported, based on validated questionnaire

^b^ Defined by WHO 2013 criteria; fasting glucose ≥5.1 mmol/l and/or 2-h plasma glucose ≥8.5 mmol/l

^c^ Collected by food frequency questionnaire in week 28 of pregnancy, and defined by cluster analysis on 55 variables of intake

### Prevalence of overweight and thinness at age 4–5 years

The prevalence of overweight was significantly higher in children of Middle East / North African origin (22.0% (14.5–29.6)) compared to children with European origin (12.8% (8.9–16.6)), while the prevalence in children with South Asian origin was significantly lower (5.2% (2.2–8.0)) (Table 2). In contrast, the prevalence of thinness was significantly higher in children with South Asian origin (26% (19.9–32.0) compared to children with European origin (10.4% (6.9–13.9)) and Middle East/North Africa origin (12.7% (6.6–18.8)).). Taking account of the South Asian’s relatively increased adiposity, we made positive adjustments of BMI. After adjustments, prevalence of thinness changed from 26 to 3.9%, and prevalence of overweight changed from 5.2 to 14.3% (Table 2).

**Table 2:** Age and prevalence of thinness, overweight and obesity in children aged 4–5 years

	Ethnic origin, n (%). Main ethnic groups					
	Total	Europe	Middle East/North Africa	South Asia unadjusted BMI	*p*-value	South Asia adjusted BMI^***^
	*n* = 570	*n* = 298	*n* = 118	*n* = 154		*n* = 154
Age in years at Child						
Health Clinic control, mean (SD)					*p* = 0.60^*^	
	4.38 (0.28)	4.37 (0.28)	4.39 (0.31)	4.40 (0.26)		
BMI category, n (%)					*p* < 0.001^**^	
Thinness	86 (15.1)	31 (10.4)	15 (12.7)	40 (26.0)		6 (3.9)
Normal weight	412 (72.3)	229 (76.8)	77 (65.3)	106 (68.8)		126 (81.8)
Overweight including obesity	72 (12.6)	38 (12.8)	26 (22.0)	8 (5.2)		22 (14.3)

^*^One-way ANOVA analysis

^**^ Chi-square test

^***^positive BMI adjustments of + 1.12 kg/m2 to account for greater relative adiposity in this population

**Fig. 2:**
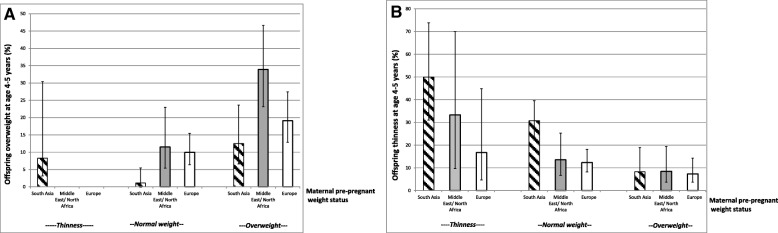
Prevalence of child overweight (**a**) and thinness (**b**) by maternal prepregnant weight status in 3 different ethnic groups

We found strong relations between mothers’ prepregnant weight status and the prevalence of overweight and thinness in the offspring. Of mothers with prepregnant overweight, 34% of Middle East/North African- and 19% of European mothers had children with overweight (Fig. 2). In contrast, 50% of South Asian mothers with thinness had children with thinness.

### Associations between maternal and early postnatal factors and overweight at age 4–5 years

Unadjusted multinomial logistic analysis (Table 3) showed that children of South Asian origin had 54% lower risk of overweight compared to European, while Middle East/North African origin had 2-fold (higher) risk. Maternal pre pregnancy overweight, low education, gestational diabetes mellitus, and child’s birthweight were also positively associated with overweight.

**Table 3:** Multinomial logistic regression for child overweight including obesity (A), and thinness (B), compared to normal weighted at age 4–5 years

Candidate factors	A. Overweight						B. Thinness					
	Unadjusted OR			Final model			Unadjusted OR			Final model		
	OR	95% CI	*p*-value	OR	95% CI	*p*-value	OR	95% CI	*p*-value	OR	95% CI	*p*-value
Ethnic origin												
Europe (reference)												
South Asia, unadjusted	0.46	0.21–1.0	0.53	0.55	0.24–1.27	0.2	2.79	0.74–4.7	< 0.001	2.20	1.25–3.87	0.006
Middle East/North Africa	2.04	1.16–3.57	0.01	1.98	1.08–3.63	0.03	0.29	0.74–20.8	0.3	1.27	0.63–2.57	0.5
Age	0.96	0.91–1.02	0.2	0.93	0.88–0.99	0.02	0.97	0.92–1.02	0.2	1.00	0.94–1.05	0.9
Prepregnant BMI, categorized												
Normal weight (reference)												
Underweight	0.44	0.06–3.40	0.4	0.45	0.06–3.72	0.5	1.80	0.82–4.0	0.1	1.89	0.81–4.43	0.1
Overweight including obesity	3.01	1.78–5.09	< 0.001	2.65	1.52–4.61	0.001	0.47	0.27–0.83	0.009	0.50	0.28–0.90	0.02
Parity												
Para 0 (reference)												
Para 1 or more	1.11	0.68–1.83	0.7				0.91	0.58–1.42	0.7			
Education												
University/college (reference)												
High School/Upper secondary school	1.58	0.90–1.79	0.1				1.24	0.76–2.05	0.4			
Primary school or less	2.00	1.04–4.00	0.04				1.26	0.66–2.41	0.5			
Prepregnant regular physical activity^a^												
Yes (reference)												
No	1.67	0.93–3.02	0.09				1.26	0.76–1.08	0.4			
Gestational diabetes mellitus^b^												
No (reference)												
Yes	1.69	1.01–2.84	0.047				1.09	0.67–1.78	0.7			
Cluster healthy/non-Healthy nutrition^c^												
Healthy (reference)												
Non-healthy	0.76	0.35–1.66	0.5				1.88	1.07–3.28	0.03			
Sex, child												
Male (reference)												
Female	1.52	0.92–2.52	0.1	1.73	0.99–3.01	0.05	0.74	0.47–1.16	0.2			
Birth weight (per 100 g)	1.12	1.07–1.18	< 0.001	1.11	1.05–1.18	< 0.001	0.93	0.90–0.97	0.001	0.96	0.92–1.01	0.1
Breast-feeding 3 months postpartum												
Yes (reference)												
No	1.58	0.79–3.18	0.2				1.07	0.52–2.17	0.9			

^a^ Self-reported, based on validated questionnaire

^b^ Defined by WHO 2013 criteria; fasting glucose ≥5.1 mmol/l and/or 2-h plasma glucose ≥8.5 mmol/l

^c^Collected by food frequency questionnaire in week 28 of pregnancy, and defined by cluster analysis on 55 variables of intake

In the adjusted model, Middle East/North African origin still nearly doubled the risk of being overweight (OR1.98; 95% CI:1.08–3.63). Maternal prepregnant overweight (OR 2.56; 95% CI: 1.52–4.61) and age (OR 0.93; 95% CI: 0.88–0.99), and child birthweight (OR1.11; 95% CI: 1.05–1.18) were independently and positively associated. Female sex was borderline significantly associated (OR1.73; 95% CI: 0.99–3.01). However, the negative effect estimate for South Asian origin was no longer statistically significant when adjusting for other factors. The association between maternal gestational diabetes and overweight in the offspring was more apparent in ethnic Europeans than in the ethnic minority groups. When formally testing for an interaction between ethnicity and gestational diabetes, however, the interaction was only borderline significant.

### Associations between maternal and early postnatal factors and thinness at age 4–5 years

Unadjusted multinomial logistic analysis (Table 3) showed that children with South Asian origin had a 2.8-fold higher risk of thinness compared to those with European origin. Maternal pre pregnancy overweight and the child’s birth weight were negatively associated with thinness, while unhealthy diet was positively associated. The adjusted model showed that South Asian origin still doubled the risk of thinness (OR 2.20; 95% CI: 1.25–3.87). Prepregnant maternal overweight (OR0.50; 95% CI: 0.28–0.90) remained independently negatively associated with thinness at 4–5 years.

The sensitivity logistic regression analysis using the adjusted classification of weight status for South Asians (Additional file 1) shows that the South Asian children have a lower risk of thinness (OR 0.22; 95% CI: 0.08–0.60) compared to the European. This result contradicts the logistic regression analysis using unadjusted BMI for South Asian children (Table 3). However, the factors positively related to overweight (Middle East/North African origin, maternal age, prepregnant overweight, female sex and birth weight) remain significant in the sensitivity model.

## Discussion

To the best of our knowledge, this study is the first population based study to assess both thinness and overweight in a multiethnic sample of children in Europe. We found strikingly different patterns of overweight and thinness among children of different ethnic groups at age 4–5 years. Compared to European children, children of Middle East/North African origin had almost the double risk of being overweight at 4–5 years of age. In contrast, South Asian origin almost doubled the risk of thinness. Applying newly suggested BMI adjustments in South Asian children, taking into account their relatively increased adiposity, markedly increased the prevalence of overweight and decreased the prevalence of thinness in this subgroup. Factors increasing the risk of overweight were maternal prepregnant overweight, lower maternal age, higher birthweight, and female sex. Maternal prepregnant overweight and higher birthweight reduced the risk of thinness. Our findings may be of great relevance for public health.

The overall prevalence of overweight and the prevalence in ethnic Europeans in our study were comparable with findings from another study of mainly ethnic Norwegian children. These data were collected in 2003–2006, and showed that in children aged 2–5 years, the prevalence of overweight was 12.7% [8]. Other European studies have only assessed either overweight or thinness. In two Dutch studies investigating overweight only, Turkish and Moroccan children had higher odds of being overweight compared to native Dutch children [4, 5]. In a British cross-sectional study investigating thinness only, the prevalence of thinness in British preschool children was 5.7%, while ethnic Asian children had an OR of 3.6 compared to white children [29]. The results from both these studies were in line with our findings.

In accordance with findings from other studies [5, 7, 30], maternal prepregnant overweight was a very strong predictor of childhood overweight. Nevertheless, it is largely unknown how much of this effect can be explained by a direct, causal relationship, for example mediated through maternal insulin resistance and inflammatory signals during pregnancy, and how much is mediated through genetics or shared life style factors [21].

Sex differences in childhood overweight vary in a global perspective [31]. In our study girls had more overweight than boys at age 4–5 years. Earlier Scandinavian studies show similar results at preschool age, but in older age groups there are more overweight in boys than in girls [8, 32].

Low socioeconomic status and low parental education have also been associated with childhood overweight [8, 30, 33]. We found no significant associations between maternal education and overweight at age 4–5 years. This might partly be due to lack of power, and that ethnicity also carries information about socioeconomic status [34]. However, other socioeconomic factors may be more important. A recent Swedish study showed that neighborhood purchasing power was more strongly related to overweight at age 4–5 years than parental educational levels [32]. This could be especially relevant in an immigrant population.

At the age of 4–5 years we found no association between gestational diabetes and offspring overweight. Studies investigating the possible link between gestational diabetes and offspring overweight show inconsistent results [35–38]. In a recent study from Hong Kong, maternal hyperglycemia in pregnancy was associated with adiposity in girls at age 7, but not in boys [36]. Our findings could suggest that also ethnicity may differentially affect this relation. Larger samples may be needed to study such effect modifications.

Other explanations of ethnic differences in overweight have been suggested. Perception of ideal BMI may vary between ethnic groups. Fatness may be associated with wealth, good health, beauty, strength, and happiness [39, 40]. In many countries, and in immigrant populations from Asia and the Middle East/North Africa, a shift from traditional food habits and entering a more sedentary life style has dramatically increased the prevalence of obesity and other risk factors for type 2 diabetes and cardiovascular diseases [41, 42].

The prevalence of thinness in the South Asian children at age 4–5 years in our study was significantly higher than in the other ethnic groups. Importantly, our definition of thinness is based on BMI z-score and does not necessarily reflect the children’s body composition. This was demonstrated in two UK studies showing that the adiposity levels were markedly higher in South Asian children than in white European children, despite lower BMI [20, 43]. In adulthood, the proportion of Asian people with a high risk of cardiovascular diseases and type 2 diabetes is substantial at BMIs lower than the WHO cut-off point for overweight [44]. However, estimated and suggested cut-off points for observed risk vary even between Asian populations and by outcomes [45]. As previously also shown in our cohort, neonates with South Asian ethnic origin are smaller and “thinner” at birth with smaller abdomen and less fat-free mass, but have relatively preserved fat mass [27]. This phenotype may result from in-utero programming after multigenerational malnutrition, tracks through life, and increases the risk of type 2 diabetes and cardiovascular disease [17, 18, 46]. Most of the South Asian children in our cohort had moderate thinness (clinically classified as grade 1 underweight). Hence, this is probably not a sign of illness, but rather due to lower lean mass. These children may be especially vulnerable if experiencing an increase in BMI; mainly representing an absolute and relative increase of fat tissue.

BMI underestimates total body fatness in South Asians. Positive BMI adjustments are suggested to make a more clinically relevant classification of weight class in children with South Asian origin [20] A recent UK study showed that when applying positive BMI adjustments in South Asian children living in England, the prevalence of thinness and overweight increased considerably in this group [47]. When applying the same BMI adjustments in our cohort of South Asian children, we also found marked changes in prevalence of thinness and overweight.

Our study suggests that health professionals should pay more attention to ethnic minority groups that are especially vulnerable to excess weight gain. On one hand, children of Middle East/North-African origin seem to have increased risk of developing overweight from early age. Overweight may persist into adult life and increase their risk of cardio-metabolic disease. On the other hand, children with South Asian origin have higher risk of thinness, probably reflecting low lean mass. This may increase their vulnerability to cardio-metabolic disease, especially if they experience excessive fat gain later in childhood or in adult life. Therefore, children who are thin from birth and in early childhood should receive earlier attention if they start crossing percentiles, and importantly, before they reach the “overweight threshold”. More research is needed before applying adjusted BMI classification based on ethnicity in a clinical setting. At Child Health Clinics and in general practice almost all children and pregnant women are followed over time from birth to adolescence and through pregnancies. This gives health professionals unique opportunities to promote healthy growth in children. Young girls and women in reproductive age can be targeted before, in, and in between pregnancies to prevent maternal overweight.

Strengths of the present study include the population-based cohort design, the large proportion of ethnic minorities that are often excluded in research, a very extensive and high quality data set, high attendance rate, and minor loss to follow-up. Limitations to this study should also be noted: Although anthropometric data on children were collected by trained Child Health Clinic personnel, they may be more inaccurate than if performed under standardized conditions. However, this probably applies equally to all ethnic groups. With 570 children in our cohort, power was limited, especially related to exploring potential effect modifications. Lifestyle characteristics, as diet and physical activity, are difficult to measure, and our variables may not represent all relevant variation. Due to small numbers for many countries of origin, we merged women into three larger ethnic groups. It cannot be ruled out that this may cause some bias because of within-group heterogeneity.

## Conclusion

We found strikingly different patterns of overweight and thinness among children of different ethnic groups at 4–5 years of age; a finding with a great relevance to public health. Compared to the European children in our cohort, children with Middle East/North African background had almost the double risk of being overweight at age 4–5 years. Maternal prepregnant overweight and younger age, and higher child birthweight also increased this risk. In contrast, children with South Asian origin had almost the double risk of being classified as thin. However, applying suggested BMI adjustments in South Asian children, taking into account their relatively increased adiposity, markedly increased the prevalence of overweight, and decreased the prevalence of thinness in this group. The deviant and contrasting weight development in both of our ethnic minority groups could represent an increased vulnerability to contract cardiovascular diseases and type 2 diabetes in adult life. This adds to the complexity health workers need to be aware of. More knowledge is needed on how to tailor culturally sensitive health care for mothers and their children to promote healthy growth and prevent disease over the life course.

## Additional file

Additional file 1: Multinomial logistic regression for child overweight including obesity and thinness, compared to normal weighted at age 4–5 years. Positive BMI adjustments for the South Asian children. (DOCX 26 kb)

## Abbreviations

BMI: Body mass index
CI: Confidence interval
OR: Odds ratio
