# Supplementary material for: Contrasting patterns of overweight and thinness among preschool children of different ethnic groups in Norway, and relations with maternal and early life factors
Source: BMC Public Health. 2018 Aug 23;18:1056. doi: 10.1186/s12889-018-5952-1 (PMC6108110; doi:10.1186/s12889-018-5952-1)
Supplement: Supplementary file 1 — Multinomial logistic regression for child overweight including obesity and thinness, compared to normal weighted at age 4–5 years. Positive BMI adjustments for the South Asian children. (DOCX 26 kb) [file 12889_2018_5952_MOESM1_ESM.docx]

Additional file

**Multinomial logistic regression for child overweight including obesity (A), and thinness (B), compared to normal weighted at age 4-5 years. Positive BMI adjustments for the South Asian children**

|  | | | | | |  |  |  |  |  |  |  |
| --- | --- | --- | --- | --- | --- | --- | --- | --- | --- | --- | --- | --- |
|  | | |  |  |  |  |  |  |  |  |  |  |
|  |  |  |  |  |  |  |  |  |  |  |  |  |
|  |  |  |  |  |  |  |  |  |  |  |  |  |
| Candidate factors | **A. Overweight** | |  |  |  |  | **B. Thinness** | |  |  |  |  |
|  | Unadjusted OR | | | Final model | | | Unadjusted OR | | |  | Final model | |
|  | OR | 95 % CI | p-value | OR | 95 % CI | p-value | OR | 95 % CI | p-value | OR | 95 % CI | p-value |
| Ethnic origin |  |  |  |  |  |  |  |  |  |  |  |  |
| Europe (reference) | 1.00 |  |  |  |  |  | 1.00 |  |  |  |  |  |
| South Asia, adjusted* | 1.05 | 0.60-1.86 | 0.8 | 1.38 | 0.80-2.72 | 0.3 | 0.35 | 0.14-0.87 | **0.02** | 0.22 | 0.08-0.6 | **0.004** |
| Middle East / North Africa | 2.04 | 1.16-3.57 | **0.01** | 1.96 | 1.08-3.56 | **0.03** | 1.44 | 0.74-2.81 | 0.3 | 1.20 | 0.60-2.43 | 0.6 |
| Age | 0.97 | 0.92-1.01 | 0.2 | 0.94 | 0.89-0.99 | **0.04** | 0.98 | 0.93-1.04 | 0.6 |  |  |  |
| pre-pregnant BMI, cathegorized |  |  |  |  |  |  | 1.00 |  |  |  |  |  |
| Normal weight (reference) |  |  |  |  |  |  |  |  |  |  |  |  |
| Underweight | 0.71 | 0.16-3.78 | 0.7 | 0.71 | 0.16-3.23 | 0.7 | 2.28 | 0.86-6.07 | 0.1 | 2.41 | 0.87-6.70 | 0.09 |
| Overweight inkl. obesity | 2.70 | 1.66-4.39 | **<0.001** | 2.42 | 1.39-3.78 | **0.001** | 0.78 | 0.40-1.51 | 0.5 | 0.74 | 0.38-1.46 | 0.4 |
| Parity |  |  |  |  |  |  |  |  |  |  |  |  |
| Para 0 (reference) |  |  |  |  |  |  | 1.00 |  |  |  |  |  |
| Para 1 or more | 1.10 | 0.69-1.75 | 0.7 |  |  |  | 0.75 | 0.42-1.33 | 0.3 |  |  |  |
| Education |  |  |  |  |  |  |  |  |  |  |  |  |
| University / college (reference) |  |  |  |  |  |  |  |  |  |  |  |  |
| High School / Upper secondary school | 1.18 | 0.70-2.0 | 0.5 |  |  |  | 0.92 | 0.48-1.75 | 0.8 |  |  |  |
| Primary school or less | 1.69 | 0.90-.015 | 0.1 |  |  |  | 1.26 | 0.57-2.76 | 0.6 |  |  |  |
| Pre-pregnant regular physical activity** |  |  |  |  |  |  |  |  |  |  |  |  |
| Yes (reference) |  |  |  |  |  |  |  |  |  |  |  |  |
| No | 1.81 | 1.04-3.16 | **0.04** |  |  |  | 1.08 | 0.58-2.01 | 0.8 |  |  |  |
| Gestational diabetes mellitus*** |  |  |  |  |  |  |  |  |  |  |  |  |
| No (reference) |  |  |  |  |  |  |  |  |  |  |  |  |
| Yes | 1.52 | 0.94-2.47 | 0.09 |  |  |  | 0.83 | 0.44-1.59 | 0.6 |  |  |  |
| Cluster healthy / non-Healthy nutrition**** |  |  |  |  |  |  |  |  |  |  |  |  |
| Healthy (reference) |  |  |  |  |  |  |  |  |  |  |  |  |
| Non-healthy | 0.65 | 0.31-1.36 | 0.3 |  |  |  | 1.55 | 0.75-3.18 | 0.23 |  |  |  |
| Sex, child |  |  |  |  |  |  |  |  |  |  |  |  |
| Male (reference) |  |  |  |  |  |  |  |  |  |  |  |  |
| Female | 1.73 | 1.08-2.78 | **0.02** | 1.98 | 1.16-3.16 | **0.008** | 1.00 | 0.56-1.78 | 1 | 1.02 | 0.56-1.86 | 1.0 |
| Birth weight (per 100g) | 1.07 | 1.03-1.12 | **0.002** | 1.09 | 1.03-1.14 | **0.001** | 0.96 | 0.92-1.02 | 0.2 | 0.95 | 0.90-1.01 | 0.09 |
| Breast-feeding 3 months postpartum |  |  |  |  |  |  |  |  |  |  |  |  |
| Yes (reference) |  |  |  |  |  |  |  |  |  |  |  |  |
| No | 1.67 | 0.87-3.16 | 0.12 |  |  |  | 0.66 | 0.23-1.94 | 0.5 |  |  |  |
| *positive BMI adjustments of +1.12 kg/m2 to account for greater relative adiposity in the South Asian population | | | | | | | | |  |  |  |  |
| **self-reported, based on validated questionnaire | | | | | | | | | | | | |
| ***Defined by WHO 2013 criteria; fasting glucose ≥ 5.1 mmol/l and/or 2-hour plasma glucose ≥ 8.5 mmol/l. | | | | | | | | |  |  |  |  |
| ****Collected by food frequency questionnaire in week 28 of pregnancy, and defined by cluster analysis on 55 variables of intake | | | | | | | | | | | |  |
